# Application of BMD Approach to Identify Thresholds of Cadmium-Induced Renal Effect among 35 to 55 Year-Old Women in Two Cadmium Polluted Counties in China

**DOI:** 10.1371/journal.pone.0087817

**Published:** 2014-02-04

**Authors:** Qi Wang, Jia Hu, Tian-xu Han, Mei Li, Huan-hu Zhao, Jian-wei Chen, Lin-xiang Ye, Yi-kai Zhou

**Affiliations:** 1 Department of Epidemiology & Biostatistics, School of Public Health, Tongji Medical College, Huazhong University of Science & Technology, Wuhan, China; 2 MOE Key Lab of Environment and Health, Institute of Environmental Medicine, School of Public Health, Tongji Medical College, Huazhong University of Science & Technology, Wuhan, China; 3 Qiangyang Center for Disease Control and Prevention, Chengdu, China; 4 Chinese Minority Traditional Medical Center, Minzu University of China, Beijing, China; National Centre for Scientific Research “Demokritos”, Greece

## Abstract

**Background:**

Cadmium (Cd) is a heavy metal that can cause renal tubular dysfunction in humans. Women are among the high-risk group for Cd health effects. Determining the thresholds of Cd-induced renal effects is important. Thus, in this article, we aimed to identify the benchmark dose (BMD) and its low limit (BMDL) levels as the Cd thresholds for Chinese women.

**Methods:**

Epidemiologic investigation was performed in county A and county B to obtain data on Cd exposure and its renal effect on respondents. Levels of Cd (UCd), β_2_-microglobulin (UB2M), and N-acetyl-β-D-glucosaminidase (UNAG) were measured in morning urine samples. The BMD approach was mainly performed.

**Results:**

Results of the BMD approach were similar whether the method was conducted for the two sets of data (collected in CA and CB, respectively) separately or cooperatively. The BMD/BMDL values of UCd for all subjects were 1.07/0.44 and 2.12/0.53 µg/g cr based on UB2M and UNAG, respectively, given a predetermined BMR of 0.05.

**Conclusions:**

The presented thresholds of Cd-induced renal effects (i.e., the BMDLs of UCd) are close to the counterpart values reported in Japan, Sweden and Belguim.

## Background

Cadmium (Cd) is a heavy metal typically acquired through environmental and occupational exposures. The general population is exposed to Cd mainly through diet and tobacco smoking [1], [2]. However, Cd exposures attributed to past and present emissions from non-ferrous industries, waste incineration, use of Cd-containing phosphate fertilizers and sewage sludge, and the burning of fossil fuels exacerbate human exposure to Cd [1]. Long-term environmental Cd exposures at low levels may result in Cd accumulation in the human body, especially in the liver and kidneys, because of the long biological half-life of Cd, which induces glomerular and tubular dysfunctions as well as osteoporosis [3], [4], [5].

Women reportedly have a higher rate of Cd absorption in the digestive tract than men [6]. This finding is possibly associated with the iron deficiency in women caused by menstruation, pregnancy, and breast-feeding [7], [8], [9], [10], [11], [12], [13]. Thus, women have a greater risk of developing cadmium toxicity than men [14]. Staessen et al. and Schutte et al. showed that despite renal tubular dysfunctions, low-level environmental Cd exposure promotes bone resorption and osteoporosis, probably leading to a higher risk of fractures, especially in postmenopausal women [3], [15].

The benchmark dose (BMD) first defined by Crump is a statistical lower confidence limit to the dose that increases the predetermined response rate defining benchmark dose response (BMR, e.g., 1% to 10%) [16]. The value corresponding to its lower 95% confidence interval (BMDL) can be used in evaluating the dose–response relationship as a replacement for the no observed adverse effect level (NOAEL) or the lowest observable adverse effect level (LOAEL) [17]. The BMD approach has been widely used to explore the thresholds of either environmental or occupational Cd exposures for Cd-induced kidney effects worldwide [18], [19], [20], [21]. However, epidemiological evidence of such exposures applicable to Chinese populations, especially women who are among the high-risk groups for environmental Cd exposure, remains inadequate.

In this article, women aged 35 to 55 years living in Cd-polluted areas were recruited to study the relationship of Cd contents with levels of β_2_-microglobulin (UB2M) and N-acetyl-β-D-glucosaminidase (UNAG) in urine and to identify the BMD and BMDL of urinary Cd (UCd) levels corresponding to predetermined BMR as 5% and 10% of the two mentioned renal tubular dysfunction biomarkers using the BMD method.

## Materials and Methods

Our research was approved by the Ethics Committee of Tongji Medical College, Huazhong University of Science and Technology. And all participants provide their written informed consent to participate in this study. This article was based on the data collected through two epidemiological investigations: one was launched in county A (CA) in 2006 and the other was launched in county B (CB) in 2011.

### Study Area

The two counties studied in our research are A (abbreviated as CA) and B (abbreviated as CB), located in the south and central of China. A non-ferrous smelter was established in CA in 1960. Total of three villages were selected. Two of the three are approximately 2 km to 4 km from the smelter on the leeward. And the third is a comparable village approximately 30 km from the smelter opposite the leeward. A smelter that started in 1980 was located in CB. This smelter was the biggest in the area, with an annual copper production of 400,000 tons. In the past 20 years, industrial wastewater had been used for irrigations of local farmland, leading to severe Cd-polluted soils and Cd-poisoned rice. Three villages approximately 2 km to 3 km from the copper smelter on the leeward and two comparable villages approximately 10 km from the smelter opposite the leeward were selected.

### Study Population

The subjects who participated in our research were healthy females aged 35 to 55 years, consisting of 209 women enrolled from CA and 269 women from CB. All of the subjects have lived in the studied areas for at least five years and mainly subsisted on local-grown crops. They mainly engaged in farming for occupation. Those with possible occupational Cd (or other toxic metals such as lead and mercury) exposures were excluded. Smokers were also excluded. A self-made questionnaire was used to collect data on the demographics of all the participants.

### Sample Collection and Analysis

Samples of morning urine (the first sample after the first-of-morning void) (50 ml to 100 ml) were collected from all the subjects and stored frozen at −80°C until analysis. The samples were collected in 250 ml polyethylene bottles soaked in 3 mol/l nitric acid for 16 h and rinsed in deionised water. UCd was examined using graphite-furnace atomic absorption spectrometry (Varian, USA). The detection limit of Cd in urine was 0.05 µg/L and no subject had values below the detection limit. The relative standard deviation (RSD) of precision test was 4.5–7.9% (n = 5), and the recovery of standard addition is 94.8–98.3%. UB2M content was measured through radioimmunoassay. UB2M kits were purchased from the Chinese Academy of Sciences. UNAG was determined as described by Tucker et al. [22]. Substrate and standard were purchased from Sigma. The amount of urinary creatinine was measured using the Jaffe reaction method [23]. Concentrations of urinary substances were expressed in corrected creatinine units (/g cr). Levels of UCd and UB2M were expressed in µg/g cr, and UNAG was expressed in U/g cr. The accuracy of each analysis was evaluated using duplicate measurements. All samples were tested in duplicate.

### Statistical Analyses

SPSS version 15.0 was used to perform the statistical analyses. The levels of the three urinary cadmium substances were confirmed to fit a log-normal distribution. Thus, geometric mean (GM) and geometric standard deviation (GSD) were used to represent their distributions. Chi-square analysis and student’s *t* test were the statistical methods used. The significance level was set at *P*<0.05.

The BMD approach, first presented by Crump (1984), was also utilized with the BMD Software (version 2.0) developed by the U.S. Environmental Protection Agency (EPA) with predetermined BMRs of 0.05 and 0.10. According to the guidelines for the BMD method of the EPA, the mathematical model with the lowest Akaike’s Information Criterion (AIC) and corresponding *P* value of 0.10 or above was the best-fit model [24]. We defined the normal cut-off point based on the 95^th^ or 90^th^ percentile. If the value found was higher than the normal cut-off points, we defined the renal function as abnormal (positive).

## Results

### Levels of UCd, UB2M, and UNAG

According to our research, subjects living in the leeward direction of the smelter had significantly higher urinary Cd levels compared to those in the opposite leeward direction in both CA and CB. So we mentioned the former who had higher Cd exposures as the high Cd-exposure group and the latter the low Cd-exposure group. The mean (SD) age was 44.8 years (5.72) and 44.1 years (5.90) for the CA and CB subjects, respectively, with no significant difference (*P = *0.191). The age distribution results of the subjects are shown in Table 1, suggesting non-significant differences of age distributions grouped by Cd exposure status among subjects in both counties.

**Table 1:** Age distribution of subjects enrolled from CA and CB.

		No. (%) of subjects			Statistics
County	Cd-exposure level	35 yrs to 44 yrs	45 yrs to 55 yrs	Total	
A	Low^a^	62 (29.7)	47 (22.5)	109 (52.2)	*χ^2^* = 1.301, *P* = 0.254
	High^b^	49 (23.4)	51 (24.4)	100 (47.8)	
	Total	111 (53.1)	98 (46.9)	209 (100.0)	
B	Low^c^	64 (23.8)	63 (23.4)	127 (47.2)	*χ^2^* = 0.158, *P* = 0.691
	High^d^	75 (27.9)	67 (24.9)	142 (52.8)	
	Total	139 (51.7)	130 (48.3)	269 (100.0)	

a Indicated the Cd exposure level of subjects enrolled in the selected village about 30 km far from the smelter opposite the leeward in CA.

b Indicated the Cd exposure level of subjects enrolled in the two selected villages about 2 km to 4 km far from the smelter on the leeward in CA.

c Indicated the Cd exposure level of subjects enrolled in the two selected villages about 10 km far from the copper smelter opposite the leeward in CB.

d Indicated the Cd exposure level of subjects enrolled in the three selected villages about 2 km to 3 km far from the copper smelter on the leeward in CB.

The GMs (GSDs) of UCd levels, UB2M concentrations, and UNAG activities arranged by age group are shown in Table 2. The GMs (GSDs) of the three Cd biomarkers were 3.00 µg/g cr (3.323), 920.48 µg/g cr (2.446), and 19.43 U/g cr (4.015) for the CA subjects, and 4.73 µg/g cr (2.882), 473.76 µg/g cr (3.322), and 3.81 U/g cr (2.121) for the CB subjects. With adjusted Cd exposure status, subjects aged 45 to 55 years were found to have modestly higher levels of UCd, UB2M, and UNAG than those who were 35 to 44 years old in both counties. However, only a few differences among them reached the significance level of 0.05.

**Table 2:** GMs (GSDs) of levels of the three studied urinary substances of the CA and CB subjects.

		Cd-exposure level in CA			Cd-exposure level in CB		
Variables	Age (yrs.)	Low^a^	High^b^	Total	Low^c^	High^d^	Total
UCd (µg/g cr)	35 to 44	1.2 (2.34)e, f	7.4(2.47)	2.7(3.51)	2.2 (2.52)e, f	6.9(2.50)	4.1(2.94)^b^
	45 to 55	1.7(2.58)^e^	6.4 (2.44)	3.4(3.09)	3.2(2.22)^e^	9.2(2.58)	5.5(2.78)
	Total	1.4 (2.48)^e^	6.9 (2.45)	3.0(3.32)	2.7 (2.41)^e^	7.9(2.56)	4.7(2.88)
UB2M (µg/g cr)	35 to 44	637.3 (2.39)^e^	1184.5(2.44)	837.9(2.54)	345.9(3.20)^f^	437.3(2.61)	392.5(3.70)^f^
	45 to 55	789.6(2.35)^e^	1300.9(2.15)	1023.8(2.32)	559.2(2.28)	598.7(3.38)	579.2(2.84)
	Total	699.0 (2.38)^e^	1242.5(2.29)	920.5(2.45)	439.0(3.70)	507.2(2.99)	473.8(3.32)
UNAG (U/g cr)	35 to 44	16.7(3.82)	17.2 (3.97)	16.9(3.87)	2.7 (2.05)e, f	4.8(2.02)	3.6(2.13)
	45 to 55	26.4 (4.56)	19.8 (3.78)	22.7(4.15)	3.7(2.11)	4.5(2.10)	4.1(2.11)
	Total	20.3(4.18)	18.5(3.86)	19.4(4.02)	3.1(2.11)^a^	4.5(2.05)	3.8 (2.12)

a Indicated the Cd exposure level of subjects enrolled in the selected village about 30 km far from the smelter opposite the leeward in CA.

b Indicated the Cd exposure level of subjects enrolled in the two selected villages about 2 km to 4 km far from the smelter on the leeward in CA.

c Indicated the Cd exposure level of subjects enrolled in the two selected villages about 10 km far from the copper smelter opposite the leeward in CB.

d Indicated the Cd exposure level of subjects enrolled in the three selected villages about 2 km to 3 km far from the copper smelter on the leeward in CB.

e Compared with the counterpart value of subjects with high-level Cd exposures, *P*<0.01 (by using students’ *t* test).

f Compared with the counterpart value of subjects aged 45 years to 55 years, *P*<0.05 (by using students’ *t* test).

### BMD and BMDL Values of UCd Based on UB2M and UNAG

In this article, BMD approach was performed in two ways. First, the representative 90^th^ percentile of UB2M and UNAG of the low Cd-exposure group were regarded as the thresholds for hyperB2Muria and hyperNAGuria, respectively. The BMD approach was separately conducted using data collected from the two mentioned epidemiologic investigations. Prevalence of hyperB2Muria and hyperNAGuria corresponding to UCd intervals of the CA and CB subjects are presented in Table 3. In the case of the 95^th^ percentile as the assumed cut-off point, few subjects had levels of UB2M (n = 13) or UNAG (n = 5) over the thresholds. Thus, the 90^th^ percentile values were selected as assumed cut-off values instead. Second, the corresponding 95^th^ percentile values of UB2M and UNAG of subjects with UCd contents below 2.0 µg/g cr were regarded as the thresholds for hyperB2Muria and hyperNAGuria, respectively. Considering the significant difference of UCd levels of the low Cd-exposure groups in CA and CB (*t* = 5.529, *P*<0.001), we did not select the corresponding 95^th^ percentile values as cut-off points. Moreover, according to our statistical analyses, the mean UCd levels were not significantly different (*t* = 0.763, *P* = 0.447) in the subjects with UCd levels below 2.0 µg/g cr. Thus, we regarded them as the control group. To be honest, this control was not a real control but a comparatively lower level of Cd-exposed group, for the GM of UCd concentrations (range: 0.15 to 1.99 µg/g cr) was 0.99 µg/g cr, which is higher than the reported values in some European populations [21], [25], [26]. The thresholds of hyperB2Muria and hyperNAGuria were separately calculated for the CA and CB subjects. Then, the BMD approach was performed with the combined two sets of dichotomous data. Prevalence of hyperB2Muria and hyperNAGuria corresponding to UCd intervals grouped by the 16.67^th^, 33.33^th^, 50^th^, 66.67^th^, and 83.33^th^ percentile of all subjects are presented in Table 4. Linear trend tests revealed the dose–response relationships of prevalent hyperB2Muria and hyperNAGuria with UCd (Tables 3 and 4).

**Table 3:** Prevalence of hyperB2Muria and hyperNAGuria corresponding to UCd intervals among subjects in CA and CB^a^.

	CA				CB				
	HyperB2Muria		HyperNAGuria		HyperB2Muria			HyperNAGuria	
UCd intervals (µg/g cr)	+/−	%	+/−	%	+/−	%		+/−	%
<2.00	3/82	3.53	3/82	3.53	2/48	4.00		3/47	6.00
2.01–4.00	4/41	8.89	4/41	8.89	5/64	7.25		4/65	5.80
4.01–10.00	13/29	30.95	8/34	19.05	13/75	14.77		19/69	21.59
>10.00	13/24	35.14	6/31	16.22	16/46	25.81		22/40	35.48
Total	33/176	15.79	21/188	10.05	36/233		13.38	48/221	17.84
Linear trend test	*χ^2^* = 26.782, *P*<0.001		*χ^2^* = 7.851, *P* = 0.005		*χ^2^* = 13.544, *P*<0.001			*χ^2^* = 22.778, *P*<0.001	

a Corresponding 90^th^ percentiles of UB2M (i.e. 1948.8 µg/g cr and 1608.7 µg/g cr) and UNAG (i.e. 90.2 U/g cr and 6.8 U/g cr) among the low-Cd exposed subjects were adopted as the thresholds for hyperB2Muria and hyperNAGuria in CA and CB, respectively. And BMD approach was used for the county-by-county data to generate BMD/BMDL values.

**Table 4:** Prevalence of hyperB2Muria and hyperNAGuria corresponding to UCd intervals grouped by the 16.67^th^ (1.26), 33.33^th^ (2.36), 50.00^th^ (3.78), 66.67^th^ (6.39), and 83.33^th^ (11.99) percentiles among all subjects a.

UCd (µg/g cr)		HyperB2Muria		HyperNAGuria	
Rang	GM	+/−	%	+/−	%
<1.26	0.70	3/76	3.80	3/76	3.80
1.27–2.36	1.75	6/74	7.50	6/74	7.50
2.37–3.78	3.03	5/75	6.25	3/77	3.75
3.79–6.39	4.91	13/67	16.25	10/70	12.50
6.40–11.99	8.53	17/63	21.25	13/67	16.25
>11.99	21.73	22/57	27.85	19/60	24.05
Total	3.88	66/412	13.81	54/424	11.30
Linear trend test		*χ^2^* = 27.944, *P*<0.001		*χ^2^* = 20.929, *P*<0.001	

a Corresponding 95^th^ percentiles of UB2M (i.e. 1198.8 µg/g cr and 849.4 µg/g cr) and UNAG (i.e. 32.1 U/g cr and 6.4 U/g cr) among the subjects with UCd of ≤2.0 µg/g cr were adopted as the thresholds for hyperB2Muria and hyperNAGuria in CA and CB, respectively. And BMD approach was used for the combined dataset to generate BMD/BMDL values.

The BMD approach was applied to present suitable models and corresponding BMDs and the low limits (BMDLs) of UCd based on UB2M and UNAG at a predetermined BMR of 0.05 or 0.10. Several models were found to fit with *P* values of >0.1. Results of the BMD approach separately conducted for CA and CB women are shown in Table 5, and those of the BMD approach conducted by combining both sets of dichotomous data are shown in Table 6. Given the limited sample size in CA and CB, the utilized BMD approach was the combination of both sets of dichotomous data. According to the guidelines of the BMD approach, the best fit model was the model with the smallest AIC [24]. The best fit model was the LogProbit model based on UB2M for the CA women, the LogLogistic model based on UB2M for the CB women, the LogProbit model based on UNAG for the CA women, and the quantal linear model based on UNAG for the CB women. In addition, the quantal linear model was likely to present fairly larger BMD and BMDL values compared with the other models.

**Table 5:** Separate BMD estimates of UCd (µg/g cr) based on UB2M (µg/g cr) and UNAG (U/g cr) for subjects in CA and CB^a^.

Variable	Model	AIC	Intercept	Slope	BMD_05_	BMDL_05_	BMD_10_	BMDL_10_	*P* d
UB2M b	**LogProbit** e	**159.86**	**−1.73**	**0.51**	**1.18**	**0.52**	**2.39**	**1.40**	**0.2113**
	LogLogistic f	160.41	−3.01	0.91	1.08	0.42	2.45	1.34	0.1561
UB2M c	LogProbit e	203.34	−1.85	0.41	1.66	0.43	4.05	1.94	0.6858
	**LogLogistic** f	**201.38**	**−3.20**	**0.74**	**1.42**	**0.35**	**3.89**	**1.92**	**0.9027**
	Quantal-Linear g	201.96		0.02	3.34	2.18	6.87	4.48	0.6681
UNAG b	**LogProbit** e	**132.66**	**−1.68**	**0.30**	**1.12**	**0.13**	**3.77**	**1.58**	**0.3478**
	LogLogistic f	132.91	−2.96	0.56	1.04	0.09	3.92	1.53	0.3042
	Quantal-Linear g	134.73		0.01	4.51	2.39	9.26	4.90	0.1113
UNAG c	LogProbit e	234.23	−2.08	0.58	2.13	0.54	3.99	1.58	0.1226
	LogLogistic f	234.38	−3.26	0.92	1.41	0.42	3.19	1.49	0.1137
	**Quantal-Linear** g	**232.86**		**0.02**	**2.14**	**1.53**	**4.41**	**3.15**	**0.2113**

a Corresponding 90^th^ percentiles of UB2M (i.e. 1948.8 µg/g cr and 1608.7 µg/g cr) and UNAG (i.e. 90.2 U/g cr and 6.8 U/g cr) among the low-Cd exposed subjects were adopted as the thresholds for hyperB2Muria and hyperNAGuria in CA and CB, respectively. And BMD approach was used for the county-by-county data to generate BMD/BMDL values.

b In CA.

c In CB.

d *P* values were obtained from the chi-square test with the Pearson goodness of fit test; if *P*>0.1 then the model is a good fit.

e LogProbit model: P[response = background+(1-background) ×CumNorm×[intercept +slope×Log(dose)].

f Loglogistic model: P[response] = background+(1-background)/[1+EXP(-intercept–slope×Log (dose)) ].

g Quantal-linear model: P[response] = background+(1-background)×[1-EXP(-slope×dose)].

**Table 6:** BMD estimates of UCd (µg/g cr) based on UB2M (µg/g cr) and UNAG (U/g cr) by conducting BMD approach with dichotomous data of all subjects^a^.

Variable	Model	AIC	Intercept	Slope	BMD_05_	BMDL_05_	BMD_10_	BMDL_10_	*P* b
UB2M	**LogProbit** c	**359.491**	**−1.67**	**0.35**	**1.07**	**0.44**	**3.02**	**1.83**	**0.6281**
	LogLogistic d	359.804	−2.92	0.64	1.00	0.36	3.09	1.80	0.5677
UNAG	**LogProbit** c	**321.496**	**−1.92**	**0.37**	**2.12**	**0.53**	**5.68**	**2.58**	**0.4057**
	LogLogistic d	321.541	−3.12	0.61	1.32	0.46	4.46	2.62	0.4022

a Corresponding 95^th^ percentiles of UB2M (i.e. 1198.8 µg/g cr and 849.4 µg/g cr) and UNAG (i.e. 32.1 U/g cr and 6.4 U/g cr) among the subjects with UCd of ≤2.0 µg/g cr were adopted as the thresholds for hyperB2Muria and hyperNAGuria in CA and CB, respectively. And BMD approach was used for the combined dataset to generate BMD/BMDL values.

b *P* values were obtained from the chi-square test with the Pearson goodness of fit test; if *P*>0.1 then the model is a good fit.

c LogProbit model: P[response = background+(1-background) ×CumNorm×[intercept +slope×Log(dose)].

d LogLogistic model: P[response] = background+(1-background)/[1+EXP(-intercept–slope×Log (dose)) ].

## Discussion

Considering that Cd could be accumulated in human bodies because of its long biological half-life in case of long-term environmental exposures, Cd storage in the body seemed to have a positive relationship with age [27]. In this article, women aged 45 to 55 years were found to have higher Cd content in their bodies than those who were 35 to 44 years old. However, significant differences in the studied urinary substances grouped by age only existed among the low-level Cd exposed subjects. Young women have been previously reported to have a larger Cd absorption rate than older ones, suggesting that young women are always at high risk of Cd exposure [28]. Considering the equality of age distribution between the subjects with environmental Cd exposures and those without, the BMD method was conducted regardless of age group [17].

According to our research, the BMD/BMDL of UCd based on UB2M were 1.18/0.13 and 2.39/1.40 µg/g cr, respectively, for a predetermined BMR of 0.05 and 0.10 in CA, and 1.42/0.35 and 3.89/1.92 µg/g cr for that in CB. Values based on UNAG were 1.12/0.13 and 3.77/1.58 µg/g cr, respectively, corresponding to a BMR of 0.05 and 0.10 in CA, and 2.14/1.53 and 4.41/3.15 µg/g cr for that in CB. Variations of BMD based on either UB2M or UNAG did not have statistical significance between the CA and CB subjects. The BMD approach was also performed by combining both sets of data from the CA and CB women. For the reason that the two investigations were not conducted simultaneously, we supposed it was not appropriate to gather the two sets of data arbitrarily for analysis. Subjects with Cd exposures of equal level (i.e. below 2 µg Cd/g cr in urine) were identified in both CA and CB, among whom corresponding 95^th^ percentiles of Cd effect biomarkers (i.e. UNAG and UB2M) were selected as the thresholds for Cd-induced renal dysfunctions. According to the predetermined thresholds the continuous variable (i.e. UNAG and UB2M) can be transformed into dichotomous data. And then pooled data could be used for BMD approach. We supposed that such approach was appropriate because the contents of urinary Cd were stable and the representative cut-off points of the renal effect biomarkers of the low-level Cd-exposed subjects in CA and CB were utilized to transform the continuous data into binary data before the BMD approach was performed. The latter application of the BMD approach presented more reliable thresholds of Cd-induced renal effect because of the larger sample size (total n = 478) than the separate applications. The corresponding BMD and BMDL values were 1.07 and 0.44 UCd/g cr based on UB2M and 2.12 and 0.53 UCd/g cr based on UNAG given a predetermined BMR of 0.05. Both applications of BMD approach revealed similar results.

Buchet et al. and Bernard et al. reported that 10% of the population exhibited renal dysfunction at urinary Cd concentrations exceeding 2–4 µg/day in the Cadmibel study [6], [29]. Järup et al. extensively reviewed the health effects of Cd and concluded that in the general population, an average urinary Cd level of 2.5 µg/g cr is related to an excess prevalence of renal tubular damage of approximately 4% [26]. Kobayashi et al. revealed the BMDLs of UCd were 1.6 µg/g and cr 3.3 µg/g cr based on UB2M in two reports for Japanes women in Cd non-polluted regions [17], [19]. According to a report of Suwazono et al., the BMDL of UCd was 0.5–0.8 µg/g cr for the 53–64 year old Swedish women [21]. That is almost the same to the counterpart value (0.53 µg/g cr) in our study for the 35–55 year old Chinese non-smoke women. We noticed that the Chinese women studied in our research had higher Cd exposure levels (with a mean UCd of 3.88 µg/g cr) compared to those in Sweden (with a mean UCd of 0.76 µg/g cr), and the UCd levels of control group were higher in the former than those in the later. However, almost the same results were obtained by both studies [21].

Recently, an updated hybrid approach has been utilized to estimate BMD and BMDL for continuous outcomes [21], [30], [31], [32], [33]. Using such method, BMD and BMDL were estimated based on a continuous exposure and a continuous effect marker without converting the data from continuous to binary form. Thus, information loss and the accompanying precision loss caused by the categorization of the participants can be avoided [31], [34], [35]. Therefore, the statistical validity and efficiency of BMD and BMDL were improved by using the hybrid approach instead of the methods involving categorization of continuous exposure and effect markers. Suwazono et al. explored the BMD and BMDL for Cd-induced renal effects in general populations and in humans with a wide range of exposure to Cd [32], [33], [36]. The BMD values of UCd obtained by using this hybrid approach were lower than the previously reported counterpart values [21]. In this article, we obtained the BMDL values of 0.52 and 0.13 µg UCd/g cr in CA and 0.35 and 1.53 µg UCd/g cr in CB based on UB2M and UNAG, respectively, with a predetermined BMR of 0.05. These results were lower than the counterpart values (0.6 µg UCd/g cr to 2.3 µg UCd/g cr based on both UB2M and UNAG for general Japanese women aged 40 to 59 years, and 3.7 µg UCd/g cr based on UB2M for Japanese women over 50 years old exposed to environmental Cd) reported by Suwazono et al. who used the hybrid approach [32], [33]. However, considering the results of the hybrid approach would be biased relevant to the variance (expressed as standard deviation) of the data and other unknown variables, such as population demographics (e.g., race, age, sex, and so on) and Cd exposure level. Further studies are necessary to compare the updated and previous BMD approaches for continuous-effect data despite the mentioned advantages of the hybrid approach [37], [38].

One of the limitations in our work was the fairly small sample size (209 in CA and 269 in CB), which may account for the broad 95% confidence intervals in the presented BMD [38]. However, this limitation could be reduced when the two sets of data are combined (total n = 478) in the BMD analysis, the procedures of which have been mentioned above. In addition, for data sets with no unexposed subjects, a dose–response model can be selected that will give as small a BMD or BMDL as desired (e.g., any value less than the lowest dose in the study) [34]. Considering the ubiquitous existence of Cd in the environment, finding a control population without any internal Cd storage in the body even in the non-Cd-polluted areas is nearly impossible [17], [32], [36], [39].

## References

[1] Hogervorst J, Plusquin M, Vangronsveld J, Nawrot T, Cuypers A, et al (2007) House dust as possible route of environmental exposure to cadmium and lead in the adult general population. Environ Res 103: 30–37. 16843453 10.1016/j.envres.2006.05.009

[2] Watanabe T, Zhang ZW, Moon CS, Shimbo S, Nakatsuka H, et al (2000) Cadmium exposure of women in general populations in Japan during 1991–1997 compared with 1977–1981. Int Arch Occup Environ Health 73: 26–34. 10672488 10.1007/pl00007934

[3] Schutte R, Nawrot TS, Richart T, Thijs L, Vanderschueren D, et al (2008) Bone resorption and environmental exposure to cadmium in women: a population study. Environ Health Perspect 116: 777–783. 18560534 10.1289/ehp.11167 PMC2430234

[4] Staessen JA, Lauwerys RR, Ide G, Roels HA, Vyncke G, et al (1994) Renal function and historical environmental cadmium pollution from zinc smelters. Lancet 343: 1523–1527. 7911869 10.1016/s0140-6736(94)92936-x

[5] Watanabe T, Iwani O, Shimbo S, Ikeda M (1993) Reduction in cadmium in blood and dietary intake among general populations in Japan. Int Arch Occup Environ Health 65: S205–208. 8406927 10.1007/BF00381342

[6] Buchet JP, Lauwerys R, Roels H, Bernard A, Bruaux P, et al (1990) Renal effects of cadmium body burden of the general population. Lancet 336: 699–702. 1975890 10.1016/0140-6736(90)92201-r

[7] Akesson A, Berglund M, Schutz A, Bjellerup P, Bremme K, et al (2002) Cadmium exposure in pregnancy and lactation in relation to iron status. Am J Public Health 92: 284–287. 11818307 10.2105/ajph.92.2.284 PMC1447058

[8] Berglund M, Akesson A, Nermell B, Vahter M (1994) Intestinal absorption of dietary cadmium in women depends on body iron stores and fiber intake. Environ Health Perspect 102: 1058–1066. 7713018 10.1289/ehp.941021058 PMC1567470

[9] Gallagher CM, Chen JJ, Kovach JS (2011) The relationship between body iron stores and blood and urine cadmium concentrations in US never-smoking, non-pregnant women aged 20–49 years. Environ Res 111: 702–707. 21507392 10.1016/j.envres.2011.03.007

[10] Jarup L, Akesson A (2009) Current status of cadmium as an environmental health problem. Toxicol Appl Pharmacol 238: 201–208. 19409405 10.1016/j.taap.2009.04.020

[11] Kippler M, Ekstrom EC, Lonnerdal B, Goessler W, Akesson A, et al (2007) Influence of iron and zinc status on cadmium accumulation in Bangladeshi women. Toxicol Appl Pharmacol 222: 221–226. 17543360 10.1016/j.taap.2007.04.009

[12] Meltzer HM, Brantsaeter AL, Borch-Iohnsen B, Ellingsen DG, Alexander J, et al (2010) Low iron stores are related to higher blood concentrations of manganese, cobalt and cadmium in non-smoking, Norwegian women in the HUNT 2 study. Environ Res 110: 497–504. 20381026 10.1016/j.envres.2010.03.006

[13] Vahter M, Berglund M, Akesson A, Liden C (2002) Metals and women’s health. Environ Res 88: 145–155. 12051792 10.1006/enrs.2002.4338

[14] Choudhury H, Harvey T, Thayer WC, Lockwood TF, Stiteler WM, et al (2001) Urinary cadmium elimination as a biomarker of exposure for evaluating a cadmium dietary exposure–biokinetics model. J Toxicol Environ Health A 63: 321–350. 11471865 10.1080/15287390152103643

[15] Staessen JA, Roels HA, Emelianov D, Kuznetsova T, Thijs L, et al (1999) Environmental exposure to cadmium, forearm bone density, and risk of fractures: prospective population study. Public Health and Environmental Exposure to Cadmium (PheeCad) Study Group. Lancet 353: 1140–1144. 10209978 10.1016/s0140-6736(98)09356-8

[16] Crump KS (1984) A new method for determining allowable daily intakes. Fundam Appl Toxicol 4: 854–871. 6510615 10.1016/0272-0590(84)90107-6

[17] Kobayashi E, Suwazono Y, Uetani M, Inaba T, Oishi M, et al (2006) Estimation of benchmark dose as the threshold levels of urinary cadmium, based on excretion of total protein, beta2-microglobulin, and N-acetyl-beta-D-glucosaminidase in cadmium nonpolluted regions in Japan. Environ Res 101: 401–406. 16436274 10.1016/j.envres.2005.12.002

[18] Kobayashi E, Suwazono Y, Dochi M, Honda R, Nishijo M, et al (2008) Estimation of benchmark doses as threshold levels of urinary cadmium, based on excretion of beta2-microglobulin in cadmium-polluted and non-polluted regions in Japan. Toxicol Lett 179: 108–112. 18515022 10.1016/j.toxlet.2008.04.013

[19] Kobayashi E, Suwazono Y, Uetani M, Inaba T, Oishi M, et al (2006) Estimation of benchmark dose for renal dysfunction in a cadmium non-polluted area in Japan. J Appl Toxicol 26: 351–355. 16791912 10.1002/jat.1147

[20] Shimizu A, Kobayashi E, Suwazono Y, Uetani M, Oishi M, et al (2006) Estimation of benchmark doses for urinary cadmium based on beta2-microglobulin excretion in cadmium-polluted regions of the Kakehashi River basin, Japan. Int J Environ Health Res 16: 329–337. 16990174 10.1080/09603120600869174

[21] Suwazono Y, Sand S, Vahter M, Filipsson AF, Skerfving S, et al (2006) Benchmark dose for cadmium-induced renal effects in humans. Environ Health Perspect 114: 1072–1076. 16835061 10.1289/ehp.9028 PMC1513341

[22] Tucker S, Boyd P, Thompson A, Price R (1975) Automated assay of N-acetyl-beta-glucosaminidase in normal and pathological human urine. Clin Chim Acta 62: 333–339. 1097134 10.1016/0009-8981(75)90245-4

[23] Bonsnes R, Taussky H (1945) On the colorimetric determination of creatinine by the Jaffe reaction. J Biol Chem 158: 581–591.

[24] EPA.US (2001) BMDS Benchmark Dose Software. For software background and tutorial see: 2001. Available: http://www.epa.gov/NCEA/bmds_training/index.htm.

[25] Akesson A, Lundh T, Vahter M, Bjellerup P, Lidfeldt J, et al (2005) Tubular and glomerular kidney effects in Swedish women with low environmental cadmium exposure. Environ Health Perspect 113: 1627–1631. 16263522 10.1289/ehp.8033 PMC1310929

[26] Jarup L, Berglund M, Elinder CG, Nordberg G, Vahter M (1998) Health effects of cadmium exposure–a review of the literature and a risk estimate. Scand J Work Environ Health 24 Suppl 11–51. 9569444

[27] Horiguchi H, Oguma E, Sasaki S, Miyamoto K, Ikeda Y, et al (2005) Environmental exposure to cadmium at a level insufficient to induce renal tubular dysfunction does not affect bone density among female Japanese farmers. Environ Res 97: 83–92. 15476737 10.1016/j.envres.2004.03.004

[28] Horiguchi H, Oguma E, Sasaki S, Miyamoto K, Ikeda Y, et al (2004) Comprehensive study of the effects of age, iron deficiency, diabetes mellitus, and cadmium burden on dietary cadmium absorption in cadmium-exposed female Japanese farmers. Toxicol Appl Pharmacol 196: 114–123. 15050413 10.1016/j.taap.2003.11.024

[29] Bernard A, Roels H, Thielemans N, Van Lierde M, Lauwerys R (1992) Assessment of the causality of the cadmium–protein relationships in the urine of the general population with reference to the Cadmibel study. IARC Sci Publ: 341–346. 1303960

[30] Crump K (1995) Calculation of benchmark doses from continuous data. Risk Anal 15: 79–89.

[31] Sand S, Victorin K, Filipsson AF (2008) The current state of knowledge on the use of the benchmark dose concept in risk assessment. J Appl Toxicol 28: 405–421. 17879232 10.1002/jat.1298

[32] Suwazono Y, Nogawa K, Uetani M, Miura K, Sakata K, et al (2011) Application of hybrid approach for estimating the benchmark dose of urinary cadmium for adverse renal effects in the general population of Japan. J Appl Toxicol 31: 89–93. 20836141 10.1002/jat.1582 PMC3010323

[33] Suwazono Y, Nogawa K, Uetani M, Nakada S, Kido T, et al (2011) Application of the hybrid approach to the benchmark dose of urinary cadmium as the reference level for renal effects in cadmium polluted and non-polluted areas in Japan. Environ Res 111: 312–314. 21190684 10.1016/j.envres.2010.11.013

[34] Crump K (2002) Critical issues in benchmark calculations from continuous data. Crit Rev Toxicol 32: 133–153. 12071571 10.1080/20024091064200

[35] Filipsson AF, Sand S, Nilsson J, Victorin K (2003) The benchmark dose method–review of available models, and recommendations for application in health risk assessment. Crit Rev Toxicol 33: 505–542. 14594105

[36] Suwazono Y, Nogawa K, Uetani M, Kido T, Nakagawa H (2011) Reassessment of the threshold of urinary cadmium by using hybrid approach in a cadmium non-polluted area in Japan. Int J Hyg Environ Health 214: 175–178. 20889376 10.1016/j.ijheh.2010.09.002

[37] Garlor DW, Jr Slikker W (2004) Role of the standard deviation in the estimation of benchmark doses with continuous data. Risk Anal 24: 1683–1687. 15660621 10.1111/j.0272-4332.2004.559_1.x

[38] Sand S, von Rosen D, Filipsson AF (2003) Benchmark calculations in risk assessment using continuous dose-response information: the influence of variance and the determination of a cut-off value. Risk Anal 23: 1059–1068. 12969419 10.1111/1539-6924.00381

[39] Wang X, Tian J (2004) Health risks related to residential exposure to cadmium in Zhenhe County, China. Arch Environ Health 59: 324–330. 16238167 10.3200/AEOH.59.6.324-330

